# Idiopathic calcinosis cutis of the scrotum: a case report and review of the literature

**DOI:** 10.1186/s13256-018-1922-6

**Published:** 2018-12-12

**Authors:** M. M. Aarif Syed, Aasiya Rajbhandari, Upama Paudel

**Affiliations:** 1 Department of Dermatology and Venereology, Institute of Medicine, Tribhuvan University, Maharajgunj, Kathmandu, Nepal; 2 Department of Pathology, Institute of Medicine, Tribhuvan University, Maharajgunj, Kathmandu, Nepal

**Keywords:** Calcinosis cutis, Scrotum, Idiopathic, Dystrophic

## Abstract

**Background:**

Abnormal deposition of calcium in the skin or subcutaneous tissue is termed calcinosis cutis. Idiopathic calcinosis cutis of the scrotum is an uncommon entity. The pathogenesis of idiopathic calcinosis cutis of the scrotum is debatable. The condition presents as several brown to yellowish nodules on the scrotum, gradually progressive, and mostly asymptomatic. Here we report a case of idiopathic calcinosis cutis of the scrotum with a brief review of the literature and a discussion on pathogenesis.

**Case presentation:**

A healthy looking, 50-year-old Nepali man presented with multiple growths on his scrotum for 15 years, which were mostly asymptomatic with an occasional complaint of itching. On physical examination, multiple pink to brown nodules ranging in size from 0.5 × 0.5 × 0.5 cm to 3 × 3 × 1 cm, which were painless and firm in consistency, were noted. On laboratory examinations the following were found to be within normal limits: serum calcium, phosphorus, parathyroid hormone, and vitamin D hormone levels; uric acid; alkaline phosphatase; and lipid profile. Based on clinical features and laboratory reports, a diagnosis of idiopathic calcinosis cutis of the scrotum was made. The nodules were excised under local anesthesia in several sittings, which gave a good cosmetic result with no evidence of recurrence in 1-year follow-up period. A histopathological examination revealed dermis with areas of fibrosis and calcification along with numerous multinucleated giant cells and an absence of any cystic structure.

**Conclusions:**

Idiopathic calcinosis cutis of the scrotum is a benign condition, which remains mostly asymptomatic. It presents as progressive multiple nodules of varying numbers and sizes. A histopathological evaluation reveals areas of calcification. The cause is either dystrophic calcification of cysts or idiopathic. Excision is the treatment of choice.

## Background

Abnormal deposition of calcium in the skin or subcutaneous tissue is termed calcinosis cutis. Skin is not a site for collection of calcium and is always a pathological phenomenon. Calcinosis cutis can involve any part of the skin. Depending on the cause, calcinosis cutis is classified into four types: dystrophic, metastatic, idiopathic, and iatrogenic [1]. Idiopathic calcinosis can present in genital skin that includes vulva, penis, or scrotum. Idiopathic calcinosis cutis of the scrotum (ICCS), also called idiopathic scrotal calcinosis, is an uncommon entity and was first described by Lewinski in 1883 [2]. In recent times, there has been a debate on the pathogenesis of scrotal calcinosis, with questions being raised on its idiopathic nature [3, 4]. The lesions are mostly asymptomatic and have great variation in sizes and numbers. Here we report a case of ICCS in a 50-year-old man with a brief review of the literature and a discussion on pathogenesis.

## Case presentation

A healthy looking, 50-year-old Nepali man came to our clinic with a complaint of multiple growths on his scrotum for 15 years. The growths started as a single lesion on the right side of his scrotum, with the gradual appearance of similar lesions on other parts. Several of these lesions coalesced at various places to form large-sized nodules. The condition was mostly asymptomatic with an occasional complaint of itching. There was no history of pain, burning sensation, trauma, ulceration, or discharge. The lesions did not interfere with urination or sexual activities. He was worried because of the increasing size of the growth and hence came to us for advice. He did not give a history of any systemic illness including metabolic, autoimmune, or malignant disorders. There was also no history of a similar complaint in his family.

**Fig. 1:**
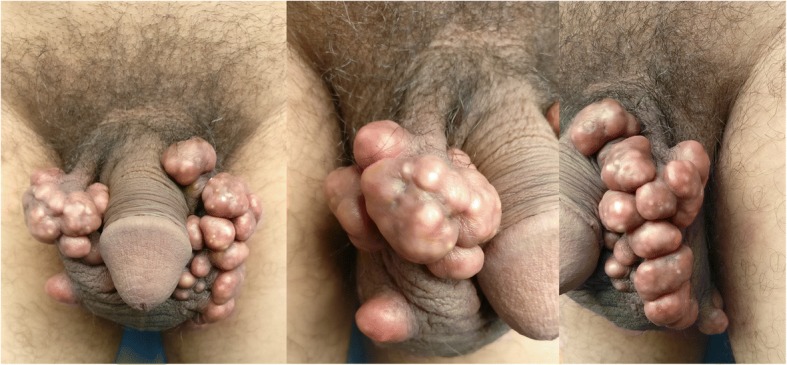
Multiple pink to brown nodules, variable in size involving a large part of the scrotum

On physical examination, multiple pink to brown nodules ranging in size from 0.5 × 0.5 × 0.5 cm to 3 × 3 × 1 cm involving almost half of his scrotum were noticed (Fig. 1). The skin over the nodules was shiny with several yellowish points indicative of underlying calcium deposition. The skin surrounding the nodules, testis, and penis was normal on palpation. The nodules were painless and firm in consistency. On laboratory examinations the following were found to be within normal limits: serum calcium, phosphorus, parathyroid hormone, and vitamin D hormone levels; uric acid; alkaline phosphatase; and lipid profile. Based on clinical features and laboratory reports, a diagnosis of ICCS was made.

**Fig. 2:**
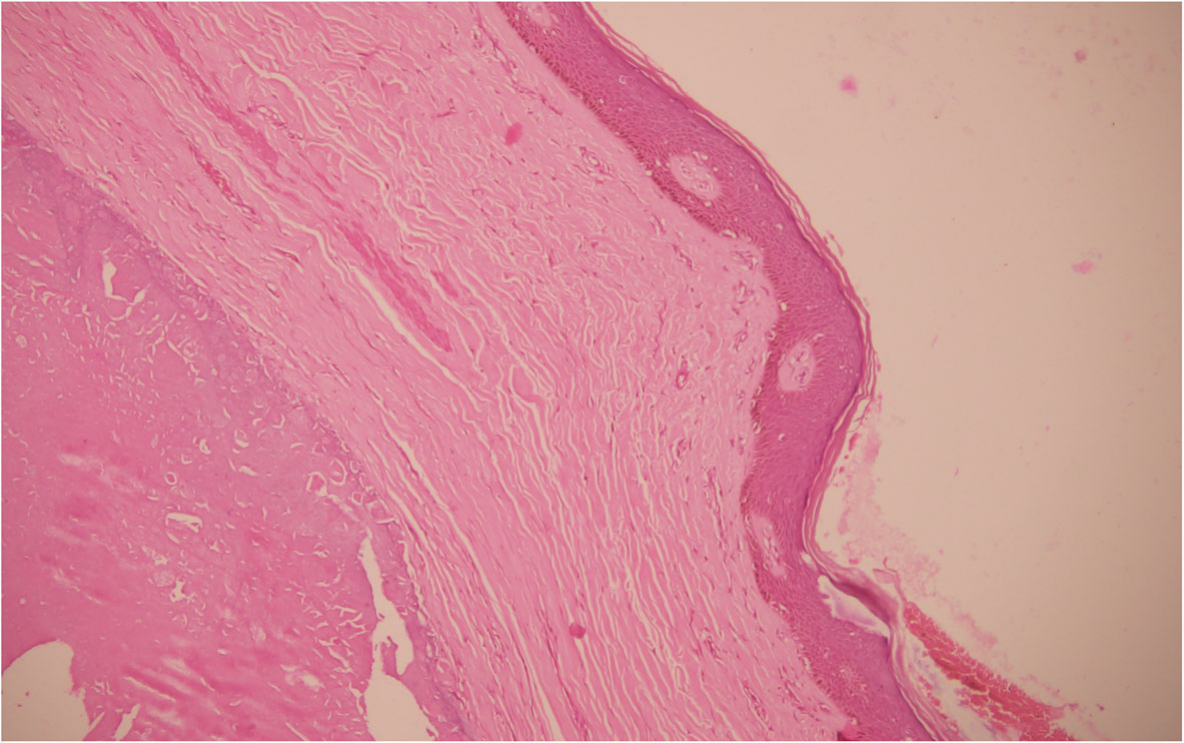
Hematoxylin and eosin stain (100 ×): keratinized stratified squamous epithelium lining the skin, with underlying dermis showing areas of fibrosis and calcification

**Fig. 3:**
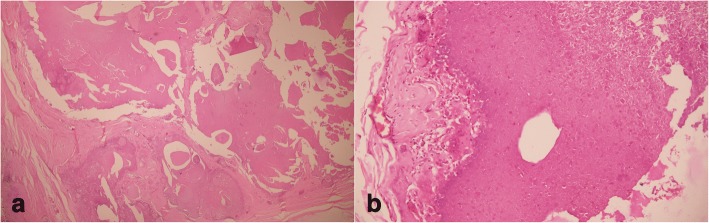
**a** and **b** Hematoxylin and eosin stain (3a is 100× while 3b is 200×): underlying dermis showing areas of fibrosis, calcification, and multinucleated giant cells

He was advised a scrotectomy under spinal anesthesia, which he refused. The nodules were excised under local anesthesia in several sittings. The skin was sutured using chromic catgut (4–0). His postoperative period was unremarkable with good cosmetic result and no evidence of recurrence in a 1-year follow-up period. The cut section of nodules showed solid white to yellow homogenous areas. Histopathological examination revealed skin tissue lined by keratinized stratified squamous epithelium. The underlying dermis had areas of fibrosis and calcification (Fig. 2). Numerous multinucleated giant cells were also seen (Fig. 3a and b). An obvious cystic structure was absent.

## Discussion

ICCS is a benign, mostly asymptomatic, condition; although it appears early, patients generally present during the third to fourth decade of life. The symptom-free nature of the disease may be the reason for delayed presentation. The disease presents as slow-growing yellowish nodules in the scrotum, single to multiple in numbers, with varying sizes. It may sometimes be associated with itching or the nodule may break down to discharge chalky material. Infection of the nodules is uncommon. Although the first case was described by Lewinski in 1883 [2], the first review was provided by Shapiro *et al.* in 1970 [5]. Lewinski is also credited with the introduction of the term idiopathic calcinosis of scrotum. The clinical features of a few case series with five or more cases reported until now are compared in Table 1. We excluded a case series that had cases of dystrophic calcinosis.

**Table 1:** Clinical features of idiopathic calcinosis cutis of the scrotum from various case reports

Authors	Shapiro *et al.* [5]	Akosa *et al.* [19]	Wright *et al*. [10]	Gormally *et al*. [6]	Baruchin *et al*. [20]	Andola *et al*. [14]
Number of cases	13*	5	9	11	5	20
Year	1970	1989	1991	1992	1998	2017
Mean age of presentation	47.3 years	39.6 years	38.66 years	**Mean age of onset: 27.4 years	36.8 years	37.2 years
Number of nodules	One to > 100	One to 36	Several	4–10 (2 cases)	One to multiple	One to multiple
Size range of nodules	1 mm to 3 cm	Not stated	A few mm to 2 cm	Up to 2 cm (2 cases)	2 mm to 1.5 cm	0.5 to 3.5 cm
Most common symptoms	Itching (5/13) Asymptomatic (2/13) Breakdown and discharge (4/13) Recurrent (1/13)	Asymptomatic (4/5) Discharge (1/5)	Asymptomatic (mostly) Itching (several) Discharge (in some)	Itching, breakdown and discharge (1/2) Asymptomatic (1/2)	Asymptomatic (4/5) Breakdown and discharge (1/5)	Asymptomatic (16/20) Breakdown and discharge (4/20)
Clinical diagnosis	Sebaceous cyst (8/13) Cyst (1/13) Not stated (3/13) Calcified fibroma (1/13)	Sebaceous cyst (5/5)	Not stated	Not stated	Not stated	Sebaceous cyst (11/20) Epidermal inclusion cyst (6/20)
Evidence of epithelial lining in histopathology	No	Yes (1/5)	No	Yes (1/11)	No	No
Evidence of calcification elsewhere in body	No	No	No	No	No	No

Veress *et al*. reported a case series with six cases from Sudan [21]. However, we could not retrieve the full paper, and hence we did not include it in our list

*Although Shapiro *et al*. mention 35 cases in their review, we have included only 13 of their own patients for analysis in the table

**Age of presentation cannot be determined

ICCS is a clinical and histological diagnosis. The size, number, skin surface, and age of onset hint toward ICCS, while confirmation is always done on histopathology. A nodular lesion in the scrotum has several differential diagnoses which include steatocystoma multiforme, angiokeratoma, lipomata, fibromata, and lymphangioma circumscriptum [6]. However, only calcified sebaceous cyst would make a difficult differential clinically. Others can be easily ruled out.

As the condition is idiopathic, the exact etiology is not known. However, there is an intense debate going on to challenge the idiopathic nature of the disease. Several authors feel that ICCS is actually a misnomer and the underlying cause is the calcification of the epidermoid or epidermal cysts of the scrotum [7–9]. In a case series of five patients with multiple calcified nodules of the scrotum, Noel *et al.* examined 15 nodules from each patient and concluded that these nodules were calcified epidermoid cysts [7]. In another study of 20 patients with scrotal calcinosis, calcification of hair follicular or epidermal cysts were the reason for calcified nodules [8]. The follicular cyst dilates, followed by calcification around and within the cyst, with eventual disappearance of epithelial lining. However, what leads to this calcification is not known. Trauma may be a triggering factor in some cases [6]. Wright *et al.* had earlier challenged this theory by using antikeratin monoclonal antibodies to find deposits of keratin in or around the calcium aggregates [10]. They failed to identify any keratin deposits, which lends support to the idiopathic nature of the disease. In recent years, Yuyucu Karabulut *et al*. further reiterated the dystrophic theory by demonstrating the presence of keratin fibers and calcium granules in the surrounding dermis [11]. It is not only the epidermoid cyst, but dystrophic calcification of eccrine glands [12] and dartos muscle [13] are also offered as alternative theories. The histopathological finding of our case did not have any evidence of epithelial lining. There were calcifications with the presence of multinucleated giant cells.

To label the condition as idiopathic, the presence of calcification elsewhere must be ruled out. A thorough biochemical and hormonal profile would help delineate the cause. If the condition is truly idiopathic, the laboratory investigations unequivocally fall within normal limits. Not one particular diagnostic modality is helpful. Fine-needle aspiration cytology, ultrasonography, and X-ray have been ordered with limited usefulness [14]. As the condition is benign and mostly asymptomatic, the treatment is for aesthetic purpose, unless the nodules start discharging or become itchy. Excision followed by scrotal reconstruction is the treatment of choice. It leaves a good cosmetic result with low chances of recurrence. Even the smallest nodule must be removed to prevent recurrence. In the genitalia, the scrotum is not the only site of idiopathic calcification. Cases of vulval [15, 16] and penile [17, 18] calcinosis have been documented.

## Conclusions

ICCS is a benign condition, which remains mostly asymptomatic. It presents as progressive multiple nodules of varying numbers and sizes. A histopathological evaluation reveals areas of calcification. The cause is either dystrophic calcification of cysts or idiopathic. Excision is the treatment of choice.

## Abbreviation

ICCS: Idiopathic calcinosis cutis of the scrotum

## References

[1] Valenzuela A, Chung L. Calcinosis: pathophysiology and management. Curr Opin Rheumatol. 2015;27(6):542–8. 10.1097/BOR.0000000000000220 26352733

[2] Lewinski HM. Lymphangiome der Haut mit Verkalklem Inhalt. Arch Pathol Anat. 1883;91:371–4.

[3] Saad AG, Zaatari GS. Scrotal calcinosis: is it idiopathic? Urology. 2001;57(2):365. 10.1016/s0090-4295(00)01007-4 11182365

[4] Dini M, Colafranceschi M. Should scrotal calcinosis still be termed idiopathic? Am J Dermatopathol. 1998;20(4):399–402. 10.1097/00000372-199808000-00014 9700381

[5] Shapiro L, Platt N, Torres-Rodriguez VM. Idiopathic calcinosis of the scrotum. Arch Dermatol. 1970;102(2):199–204. 5464321

[6] Gormally S, Dorman T, Powell FC. Calcinosis of the scrotum. Int J Dermatol. 1992;31(2):75–9. 10.1111/j.1365-4362.1992.tb03239.x 1559744

[7] Noel B, Bron C, Kunzle N, De Heller M, Panizzon RG. Multiple nodules of the scrotum: histopathological findings and surgical procedure. A study of five cases. J Eur Acad Dermatol Venereol. 2006;20(6):707–10. 10.1111/j.1468-3083.2006.01578.x 16836500

[8] Shah V, Shet T. Scrotal calcinosis results from calcification of cysts derived from hair follicles: a series of 20 cases evaluating the spectrum of changes resulting in scrotal calcinosis. Am J Dermatopathol. 2007;29(2):172–5. 10.1097/01.dad.0000246465.25986.68 17414441

[9] Song DH, Lee KH, Kang WH. Idiopathic calcinosis of the scrotum: histopathologic observations of fifty-one nodules. J Am Acad Dermatol. 1988;19(6):1095–101. 10.1016/s0190-9622(88)70278-9 2462577

[10] Wright S, Navsaria H, Leigh IM. Idiopathic scrotal calcinosis is idiopathic. J Am Acad Dermatol. 1991;24(5):727–30. 10.1016/0190-9622(91)70110-n 1714467

[11] Yuyucu Karabulut Y, Kankaya D, Senel E, Dolek Y, Uslu A, Sertcelik A. Idiopathic scrotal calcinosis: the incorrect terminology of scrotal calcinosis. G Ital Dermatol Venereol. 2015;150(5):495–9. 26333550

[12] Dare AJ, Axelsen RA. Scrotal calcinosis: origin from dystrophic calcification of eccrine duct milia. J Cutan Pathol. 1988;15(3):142–9. 10.1111/j.1600-0560.1988.tb00534.x 3294255

[13] King DT, Brosman S, Hirose FM, Gillespie LM. Idiopathic calcinosis of scrotum. Urology. 1979;14(1):92–4. 10.1016/0090-4295(79)90225-5 452234

[14] Andola S, Tandon T, Patil A. Clinical-epidemiological, cytological and histopathological study of idiopathic calcinosis cutis of the scrotum. J Clin Diagn Res. 2017;11(11):EC01–EC4.

[15] Jamaleddine FN, Salman SM, Shbaklo Z, Kibbi AG, Zaynoun S. Idiopathic vulvar calcinosis: the counterpart of idiopathic scrotal calcinosis. Cutis. 1988;41(4):273–5. 3366012

[16] Coban YK, Aytekin AH, Aydin EN. Idiopathic Calcinosis Cutis of the Vulva. Indian J Dermatol. 2013;58(6):464–6. 10.4103/0019-5154.119960 PMC3827520 24249900

[17] Cohen PR, Tschen JA. Idiopathic calcinosis cutis of the penis. J Clin Aesthet Dermatol. 2012;5(12):23–30. PMC3533325 23277801

[18] Katoh N, Okabayashi K, Wakabayashi S, Kishimoto S, Yasuno H. Dystrophic calcinosis of the penis. J Dermatol. 1993;20(2):114–7. 10.1111/j.1346-8138.1993.tb03842.x 7683026

[19] Akosa AB, Gilliland EA, Ali MH, Khoo CT. Idiopathic scrotal calcinosis: a possible aetiology reaffirmed. Br J Plast Surg. 1989;42(3):324–7. 10.1016/0007-1226(89)90155-0 2667677

[20] Baruchin AM, Baruffaldi Preis FW, Cavallini M, Ben-Dor D. Idiopathic calcinosis of the scrotum: report of five cases and review of the literature. Eur J Plast Surg. 1998;21(6):282–5.

[21] Veress B, Malik M. Idiopathic scrotal calcinosis. A report of six cases from the Sudan. East Afr Med J. 1975;52:705–10. 1222698

